# Researches on the Medical Properties and Applications of Nitrous Oxide, Protoxide of Nitrogen or Laughing Gas

**Published:** 1865-10

**Authors:** 


### RESEARCHES ON THE MEDICAL PROPERTIES AND APPLICATIONS OF NITROUS OXIDE, PROTOXIDE OF NITROGEN OR LAUGHING GAS.

BY GEO. J. ZEIGLER, M. D.

*Physician to the Philadelphia Hospital, member of the American Medical Association, member of the Academy of Natural Sciences of Philadelphia, etc., etc.*

This is a very condensed and comprehensive treatise on the properties and uses of Protoxide of Nitrogen. It is an epitome of the author's observations, researches and writings, which have been published from time to time in the various medical journal of the country.

The views and opinions contained in this work, are entitled to a very high regard, based as they are, upon researches extending through many years. Works written in this style, are for most readers, preferable to those long drawn, prolix works, in which many a time the ideas are hidden beneath a flood of words. In this utilitarian age, very few indeed are disposed to devote time and patience to the study of attenuated hypothesis. This work we regard the best compend upon the subject, and would suggest for it a careful perusal.

To those of our profession we would suggest that it may be obtained of S. S. White, or we presume at most of the dental depots.

